# Supplementary material for: SKOOTS: Skeleton‐Oriented Object Segmentation for Mitochondria in High‐Resolution Cochlear EM Datasets
Source: Adv Sci (Weinh). 2026 Mar 31;13(32):e17738. doi: 10.1002/advs.202517738 (PMC13252615; doi:10.1002/advs.202517738)
Supplement: Supplementary file 1 — Supporting File 1: advs75000‐sup‐0001‐Figures1.pdf. [file ADVS-13-e17738-s002.pdf]

SKOOTS: Skeleton-oriented object segmentation for mitochondria

Christopher J Buswinka[1,2,3], Richard T. Osgood[1,2], Hidetomi Nitta[1], Artur A. Indzhykulian[1,2,3]

1 - Eaton Peabody Laboratories, Mass Eye and Ear, Boston, MA, USA.
2 - Department of Otolaryngology, Head and Neck Surgery, Harvard Medical School, Boston, MA, USA.
3 - Speech and Hearing Biosciences and Technology graduate program, Harvard University, Cambridge, MA, USA.

Supplemental Figures

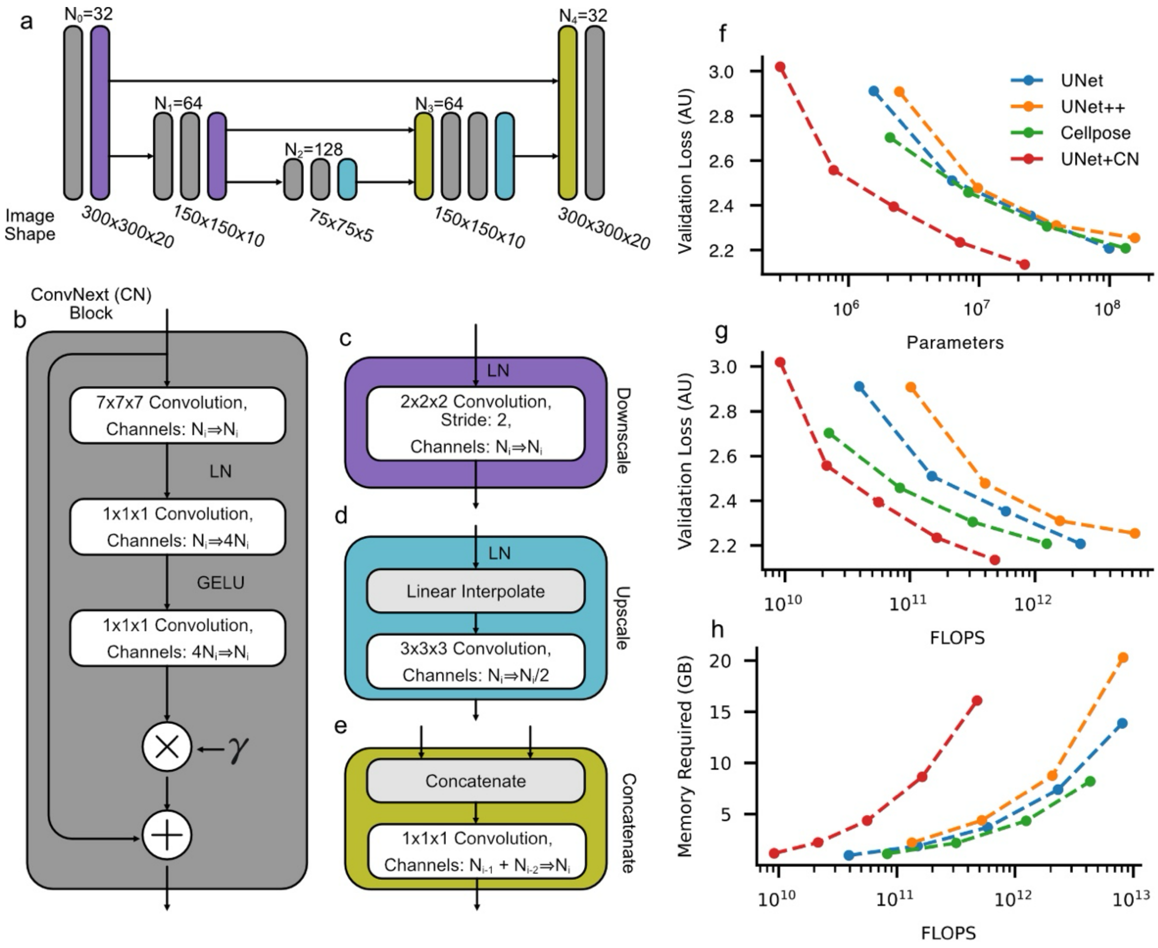

Supplemental Figure 1. UNet Architecture with ConvNext computational blocks offers superior accuracy per floating-point operations/second (FLOPS) in a semantic segmentation task. (**a**) Structure of a UNet-like model, composed of four major neural-network computational blocks, summarized in panels b-e, and color-coded across a-e for clarity. (**b**) a ConvNext residual block replaces the standard two stacked convolutions of UNet with a single convolution with increased kernel size (7x7x7) followed by two pointwise convolutions and a learnable scaling parameter (1/2×), (**c**) a downscale block composed by a 3D convolutional kernel with a 2x2x2 shape and a stride of 2, (**d**) an upscale block achieved by linear interpolation followed by convolution, and (**e**) a concatenation block. (**f-h**) Upon direct comparison, this architecture (**Red**) achieves superior performance semantic segmentation compared to currently reported best alternative architectures (UNet, UNet++ and Cellpose), with (**f**) a lower number of parameters, (**g**) a lower number of FLOPS, however, needs slightly more (**h**) video memory to process identically sized volumes when compared to alternatives.

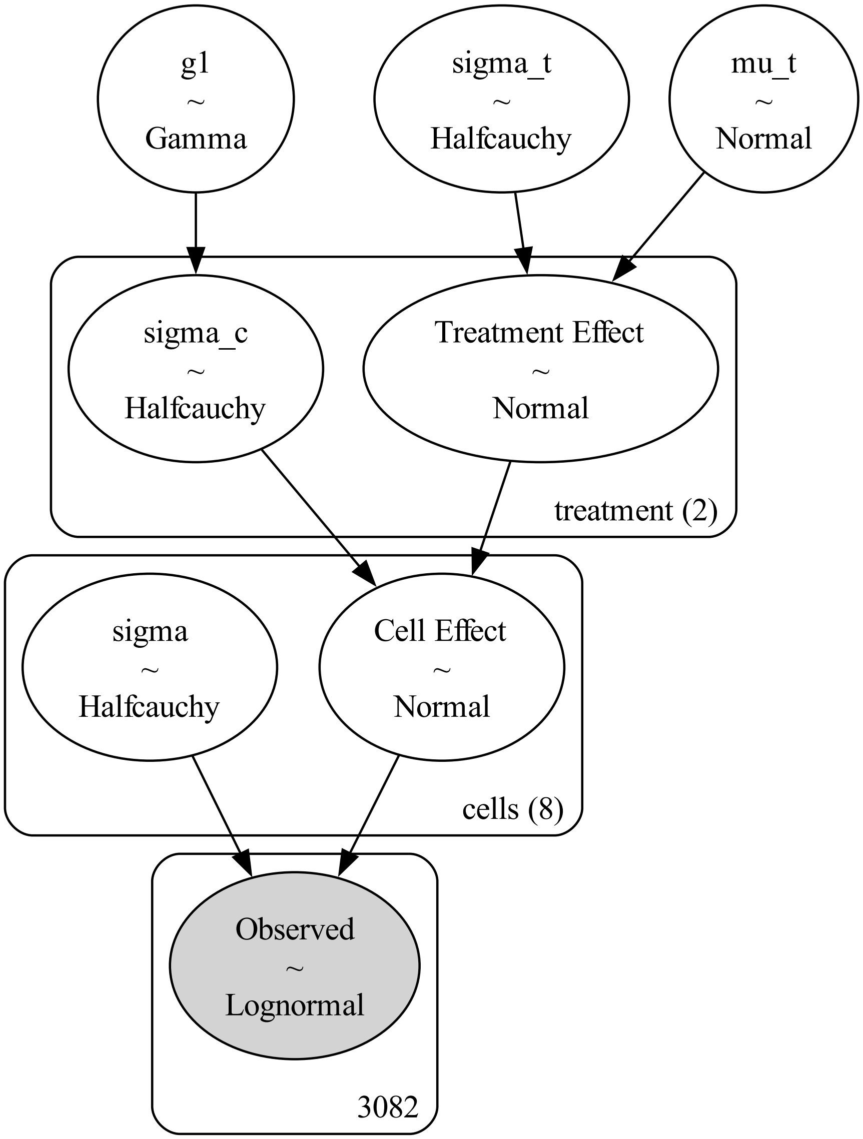

Supplemental Figure 2: **Bayesian hierarchical model for assessing treatment effects of aminoglycoside on mitochondria morphology.** All prior distributions were uninformative and did not significantly bias model results.

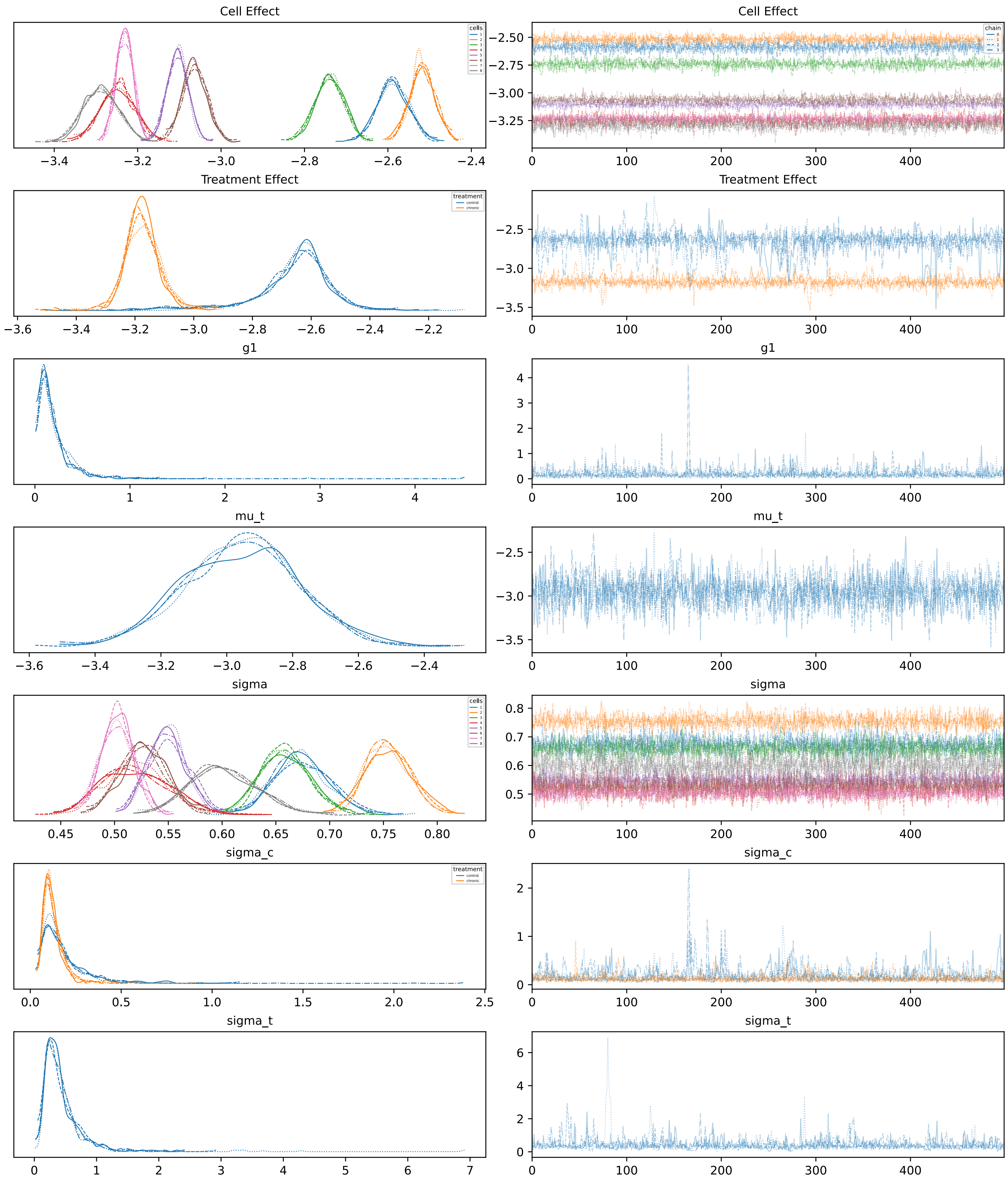

Supplemental Figure 3: Trace plot of model sampling fit on mitochondria volume. Four chains using the No U-Turn Sampler (NUTS) were sampled five hundred times to infer posterior distributions of our model. No divergences were observed and all R Hat statistics were less than one. Each line represents an independent Markov Chain Monte Carlo (MCMC) sampling chain (n=4), showing good convergence and stable behavior, suggesting reliable estimates. MCMC is a computational method that approximates complex probability distributions by generating samples through a stochastic, sequential process. Control: cells #1-#3, chronic application of 10 µM gentamicin: cells #4-#8.

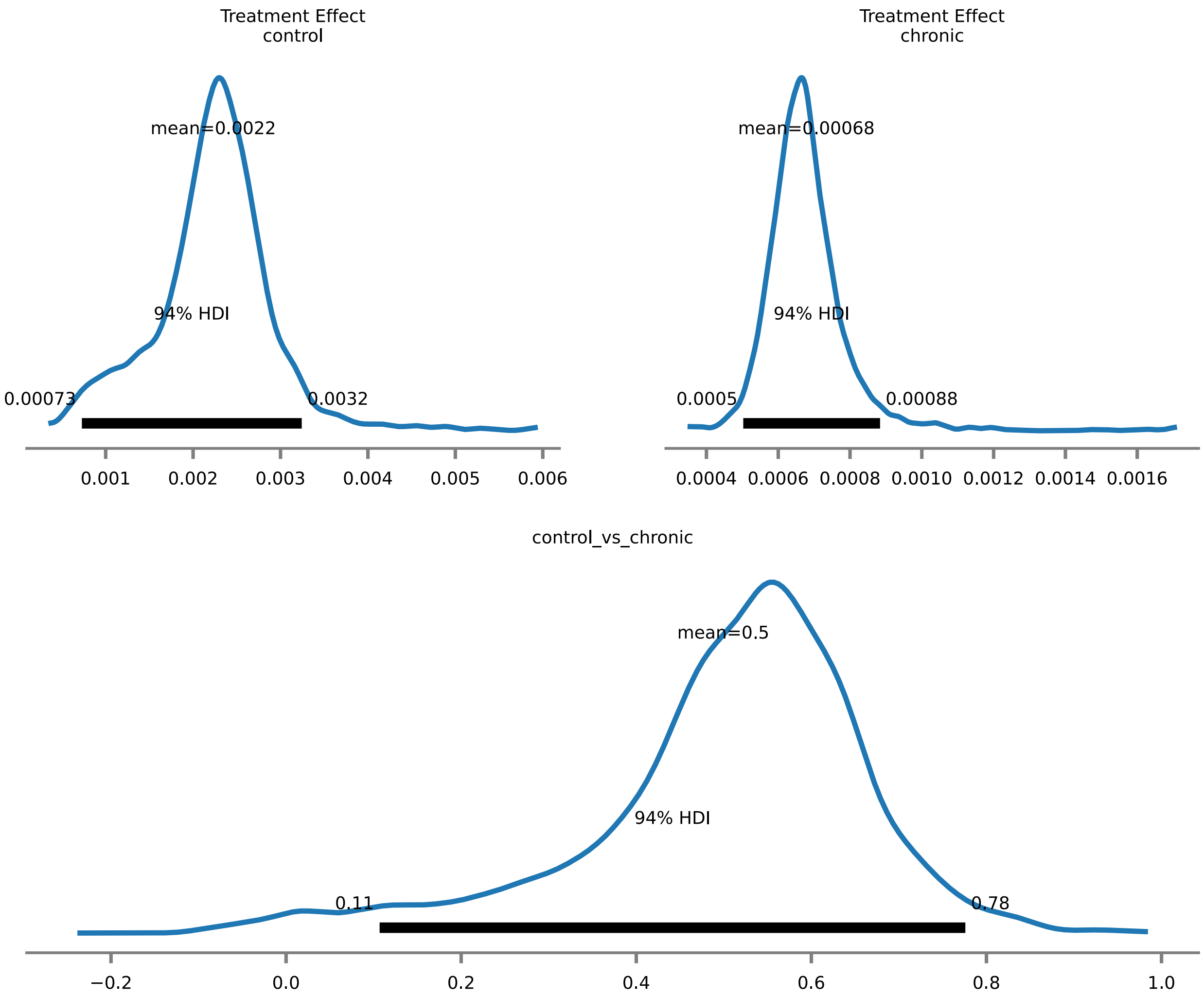

Supplemental Figure 4: Posterior predictive distributions of treatment effect of aminoglycoside treatment on mitochondrial volume. 94% credible intervals greater than zero indicate a statistically significant effect of both acute and chronically treated aminoglycoside treatment on mitochondrial volume.

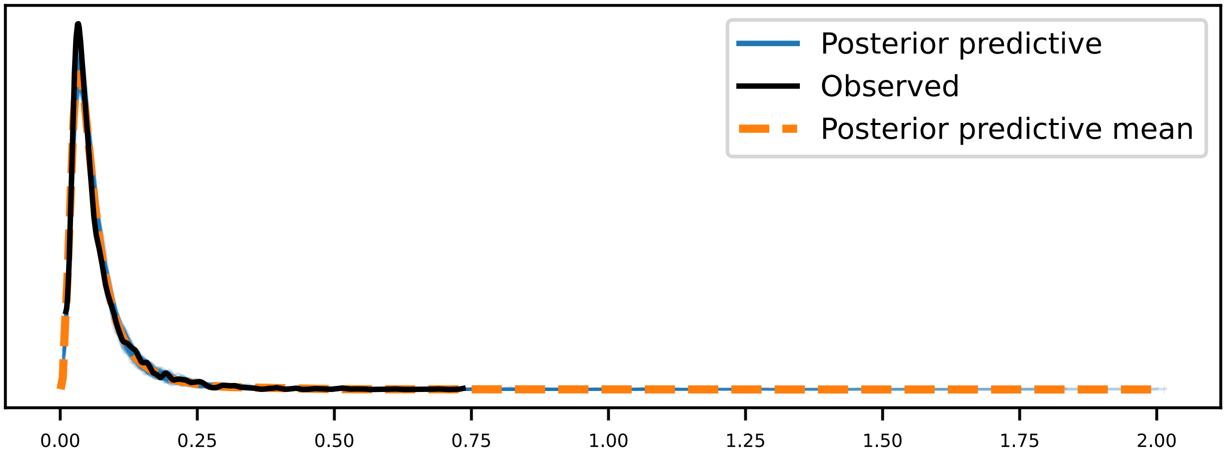

Supplemental Figure 5 Posterior predictive check of mitochondria volume indicates the model approximates the observed data well.

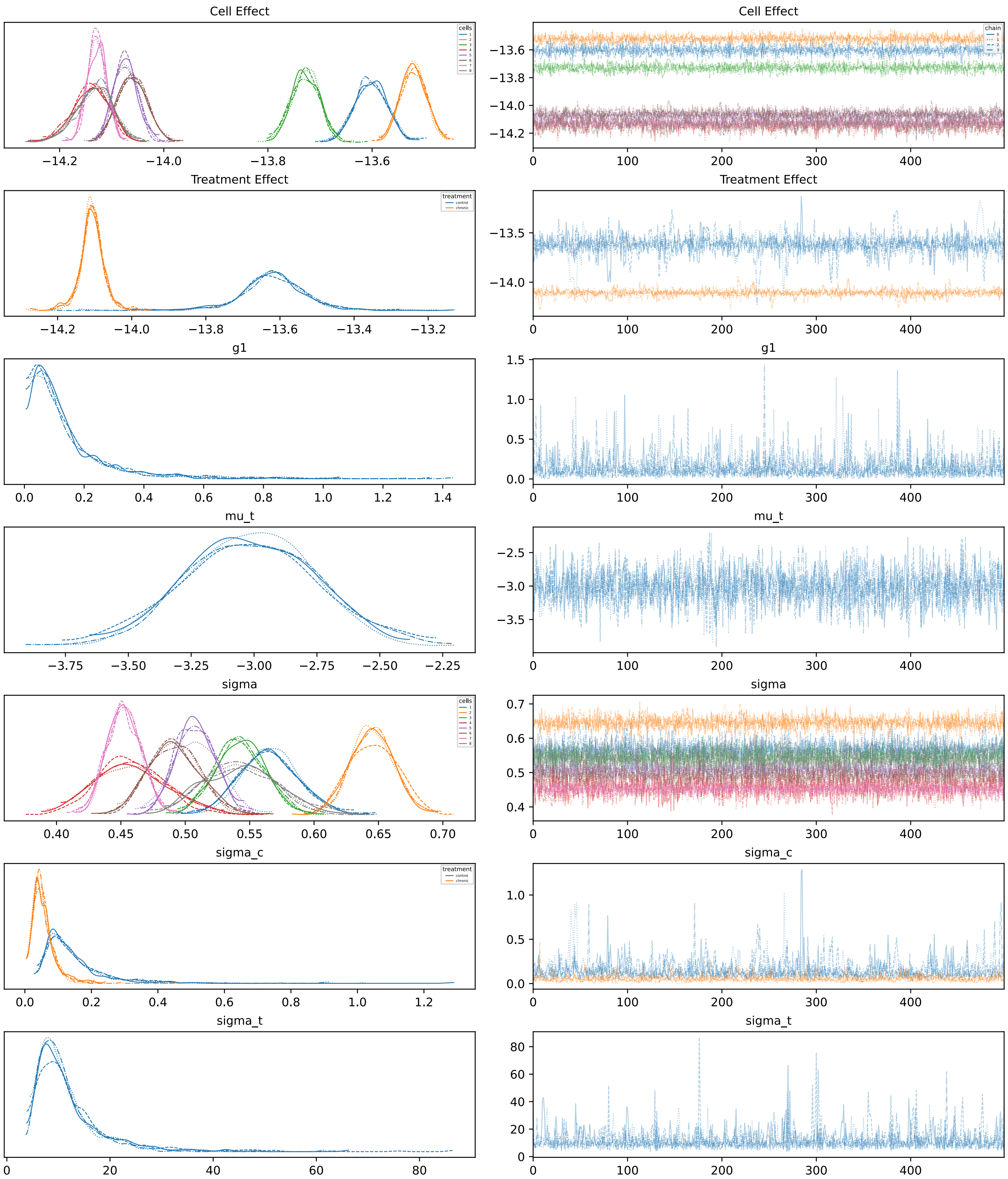

Supplemental Figure 6: Trace plot of model sampling fit on mitochondria surface area. Four chains using the No U-Turn Sampler (NUTS) were sampled five hundred times to infer posterior distributions of our model. No divergences were observed and all R Hat statistics were less than one. Each line represents an independent Markov Chain Monte Carlo (MCMC) sampling chain (n=4), showing good convergence and stable behavior, suggesting reliable estimates. Control: cells #1-#3, chronic application of 10 µM gentamicin: cells #4-#8.

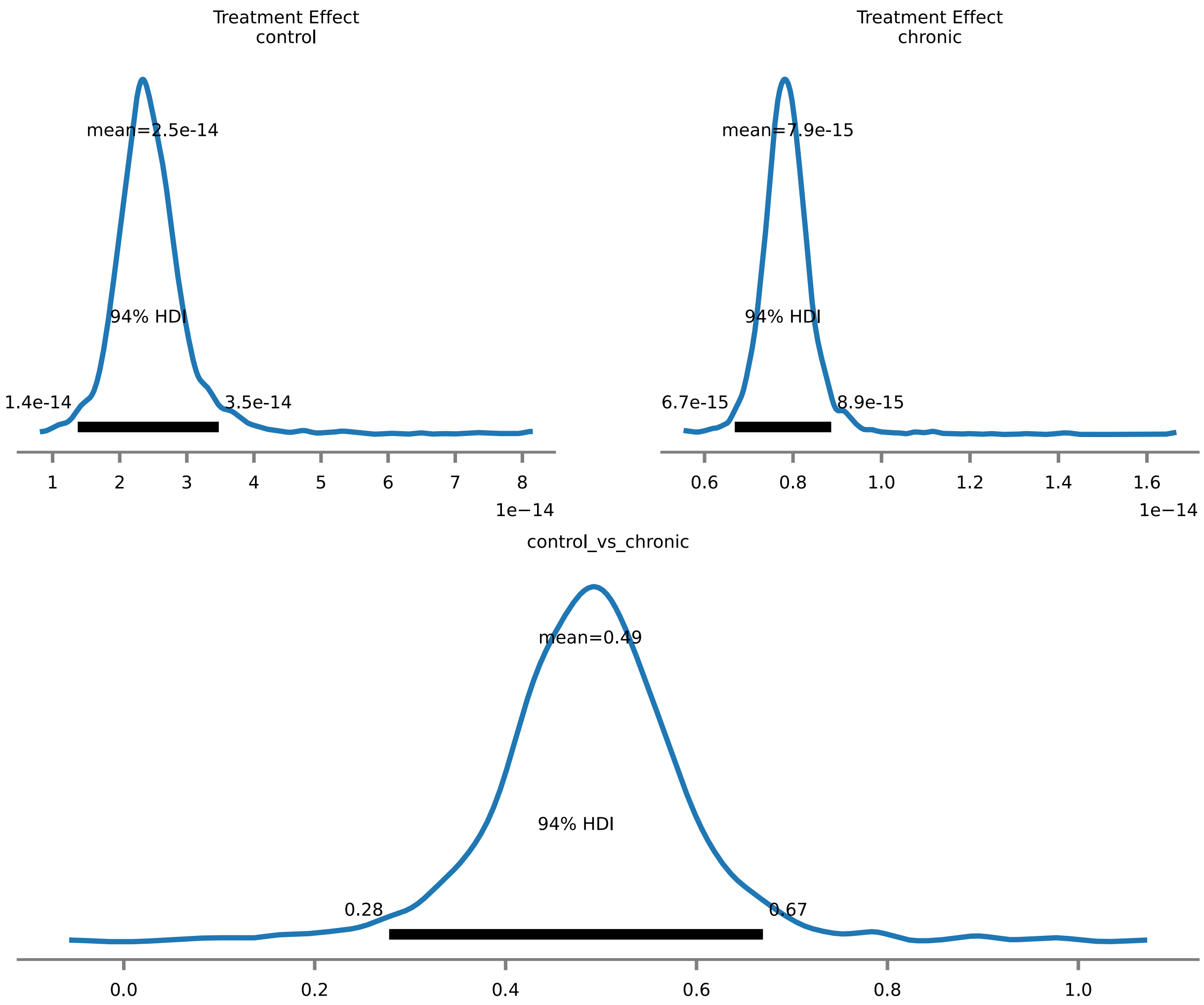

Supplemental Figure 7 Posterior predictive distributions of treatment effect of aminoglycoside treatment on mitochondrial surface area. 94% credible intervals greater than zero indicate a statistically significant effect of both acute and chronically treated aminoglycoside treatment on mitochondrial surface area.

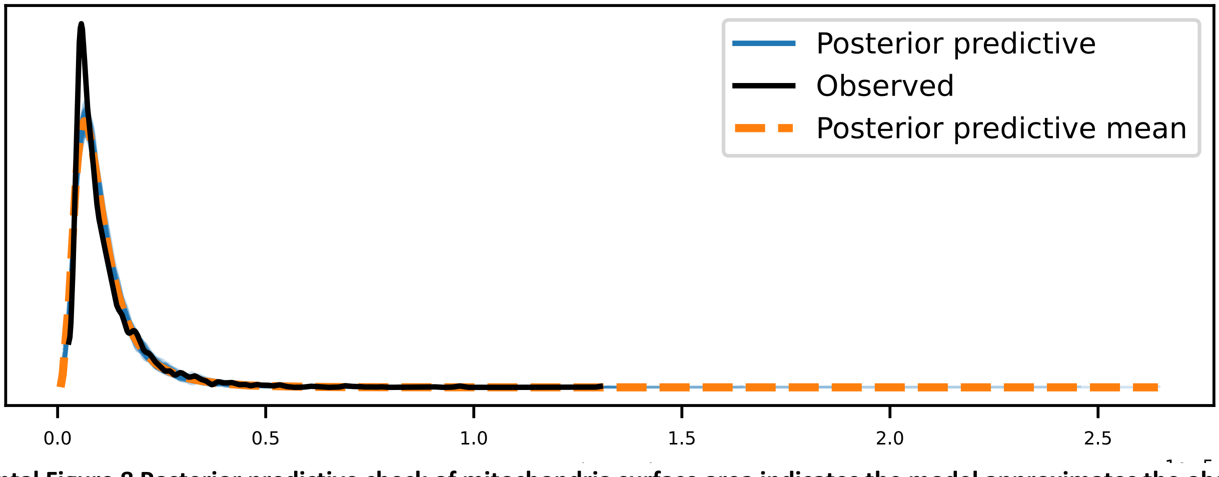

Supplemental Figure 8 Posterior predictive check of mitochondria surface area indicates the model approximates the observed data well.
